# An injectable, self-healing, electroconductive hydrogel loaded with neural stem cells and donepezil for enhancing local therapy effect of spinal cord injury

**DOI:** 10.1186/s13036-023-00368-2

**Published:** 2023-07-24

**Authors:** Tiemei Liu, Qiang Zhang, Hongru Li, Xiaoqian Cui, Zhiping Qi, Xiaoyu Yang

**Affiliations:** 1 Department of Blood Transfusion, China-Japan Union Hospital of Jilin University, 130033 Changchun, China; 2 Department of Orthopaedic Surgery, The Second Hospital of Jilin University, 130041 Changchun, China; 3 Department of Emergency and Critical Care, The Second Hospital of Jilin University, 130041 Changchun, PR China

**Keywords:** Hydrogel, Conductivity, Spinal cord injury, Neural stem cells, Donepezil

## Abstract

**Background:**

Spinal cord injury (SCI) is a serious injury with high mortality and disability rates, and there is no effective treatment at present. It has been reported that some treatments, such as drug intervention and stem cell transplantation have positive effects in promoting neurological recovery. Although those treatments are effective for nerve regeneration, many drawbacks, such as low stem cell survival rates and side effects caused by systemic medication, have limited their development. In recent years, injectable hydrogel materials have been widely used in tissue engineering due to their good biocompatibility, biodegradability, controllable properties, and low invasiveness. The treatment strategy of injectable hydrogels combined with stem cells or drugs has made some progress in SCI repair, showing the potential to overcome the drawbacks of traditional drugs and stem cell therapy.

**Methods:**

In this study, a novel injectable electroactive hydrogel (NGP) based on sodium hyaluronate oxide (SAO) and polyaniline-grafted gelatine (NH_2_-Gel-PANI) was developed as a material in which to load neural stem cells (NSCs) and donepezil (DPL) to facilitate nerve regeneration after SCI. To evaluate the potential of the prepared NGP hydrogel in SCI repair applications, the surface morphology, self-repairing properties, electrical conductivity and cytocompatibility of the resulting hydrogel were analysed. Meanwhile, we evaluated the neural repair ability of NGP hydrogels loaded with DPL and NSCs using a rat model of spinal cord injury.

**Results:**

The NGP hydrogel has a suitable pore size, good biocompatibility, excellent conductivity, and injectable and self-repairing properties, and its degradation rate matches the repair cycle of spinal cord injury. In addition, DPL could be released continuously and slowly from the NGP hydrogel; thus, the NGP hydrogel could serve as an excellent carrier for drugs and cells. The results of in vitro cell experiments showed that the NGP hydrogel had good cytocompatibility and could significantly promote the neuronal differentiation and axon growth of NSCs, and loading the hydrogel with DPL could significantly enhance this effect. More importantly, the NGP hydrogel loaded with DPL showed a significant inhibitory effect on astrocytic differentiation of NSCs in vitro. Animal experiments showed that the combination of NGP hydrogel, DPL, and NSCs had the best therapeutic effect on the recovery of motor function and nerve conduction function in rats. NGP hydrogel loaded with NSCs and DPL not only significantly increased the myelin sheath area, number of new neurons and axon area but also minimized the area of the cystic cavity and glial scar and promoted neural circuit reconstruction.

**Conclusions:**

The DPL- and NSC-laden electroactive hydrogel developed in this study is an ideal biomaterial for the treatment of traumatic spinal cord injury.

**Supplementary Information:**

The online version contains supplementary material available at 10.1186/s13036-023-00368-2.

## Introduction

Spinal cord injury (SCI) is caused by direct or immediate mechanical injury to the spinal cord, which can permanently damage the connection between the brain and peripheral organs, resulting in sensorimotor dysfunction [1, 2]. Furthermore, SCI can also cause a series of secondary diseases, such as muscle spasm, osteoporosis, urinary tract infection, bedsores and respiratory complications, which impose heavy economic costs and practical challenges on patients and can even endanger their lives. Spinal cord tissue has a poor ability to repair itself, resulting in poor treatment outcomes after spinal cord injury. There are two main reasons why spinal cord tissue regeneration is weak. First, the regenerative capacity of neurons in the central nervous system is limited [3]. Second, after spinal cord injury, the local microenvironment is inhibitory, which leads to the formation of glial scars and cavities around the lesion centre, hindering the infiltration of cells and the regeneration of axons and thereby inhibiting nerve regeneration [1]. Therefore, in the treatment of traumatic spinal cord injury, stimulation of nerve cell differentiation and neurite growth, along with inhibition of glial scar and cavity production, are the keys to effective repair of traumatic spinal cord injury.

To date, the treatment of spinal cord injury has focused on surgical decompression and stabilization. However, there is no effective clinical treatment for neurological recovery after SCI, and the repair of severe SCI remains a major clinical challenge. Over the past few decades, efforts have been made to develop treatments for nerve regeneration. Stem cells are an effective treatment for spinal cord injury because they promote nerve regeneration and provide neuroprotection [4]. Researchers have found that modulating inflammatory responses and reversing motor deficits associated with acute spinal cord injury by supplementing with neural stem cells is a reasonable and promising therapeutic strategy for spinal cord injury [5]. Unfortunately, despite extensive stem cell therapy research, one major obstacle remains: up to 90% of transplanted cells do not survive when they are passaged into neural tissue [6]. Another traditional way to promote nerve regeneration is the topical use of neuroprotective factors and anti-inflammatory drugs, which are meant to reverse the microenvironmental conditions that inhibit axonal growth. However, traditional medicine therapy is often less effective in spinal cord injury repair due to the rapid clearance of cerebrospinal fluid and impaired biological activity [7]. Furthermore, safely delivering a therapeutic dose of the drug to the spinal cord tissue is a major challenge. Many drugs exhibit negligible accumulation in central nervous system tissues after systemic administration because drugs cannot cross the blood‒spinal barrier effectively. Even if a drug can cross the blood‒brain barrier, it usually requires a high systemic drug dose to reach therapeutic levels at the site of injury, resulting in harmful side effects [8]. Therefore, there is an urgent need for a suitable drug/cell transporter to improve the therapeutic effect of spinal cord injury.

In recent years, injectable hydrogels have shown great promise in the treatment of severe spinal cord injury because they have abundant water content and are similar to natural soft tissue biomolecules. Hydrogels can provide a microenvironment that supports extracellular matrix functions such as anchoring, signal transduction, and nutrient transport [9, 10]. The tight adhesion of the hydrogel and spinal cord can relink the damaged nerve. More importantly, when used as a cell culture substrate, injectable hydrogels provide a suitable environment for cell adhesion and growth without interfering with the transport of essential nutrients, thereby improving cell retention and survival in target tissues. At the same time, injectable hydrogels can also realize the slow release of drugs in the damaged area, avoiding the problems existing in traditional drug treatment [11]. Therefore, injectable hydrogels can be used as good carriers for stem cells and drugs.

Previous studies have demonstrated that electroactive materials can accelerate the healing of injured nerves [12, 13]. The addition of conductive materials to hydrogels can effectively promote the transmission of electrical signals and has been shown to be beneficial to the proliferation and differentiation of nerve cells [14]. Furthermore, conductive hydrogels can simulate the physiological environment of electroactive tissues, regulate the spinal cord microenvironment, and reconstruct the signal transduction pathway of damaged nerves, thus promoting nerve regeneration. To date, a variety of conductive materials, such as polyaniline (PANI), polypyrrole (PPy), polythiophene (PTH), metal nanoparticles and carbon-based materials, have been introduced to construct conductive hydrogels [15–19]. Carbon-based materials mainly include carbon nanotubes (CNTs), graphene, carbon points and MXene, etc. These materials have good environmental stability, high electrical conductivity and low production cost. Many studies have mixed carbon-based materials into hydrogels to prepare conductive hydrogels and verified their application in promoting tissue repair [20, 21]. Park, J et al. added graphene oxide (GO) to gelatin methylacrylyl (GelMA) hydrogel, and the results showed that the addition of GO could significantly enhance the conductivity of the hydrogel and improve cell proliferation in the hydrogel [22]. However, carbon-based materials have certain defects in application, such as the material is easy to cause thrombosis in the body and is difficult to be metabolized by the body. Metal nanoparticles have high electrical conductivity, interesting optical and catalytic properties, and are easy to manufacture and modify. Therefore, conductive hydrogels based on metal nanomaterials have been widely studied in the field of nerve repair in recent years. Among all metal nanoparticles, silver nanoparticles (Ag) are the most widely used metal in biomaterials [23, 24]. In addition to its conductivity, Ag has strong antibacterial properties and is also approved by the FDA. In addition, gold nanoparticles are also widely used in the field of nerve repair. For example, some studies have loaded gold nanoparticles into injectable hydrogels in which the gold nanoparticles promoted nerve cell differentiation under electrical stimulation conditions and further promoted tissue repair [25]. Similarly, metal nanoparticles also have certain defects, which themselves have certain cytotoxicity and can easily lead to thrombosis. Among them, conductive polymers such as polyaniline is widely used in tissue engineering because of its reasonable structural design, good water solubility and good electrical activity. However, the poor solubility and non-degradability of polyaniline have been obstacles to its widespread application. Recently, some studies grafted polyaniline and natural polymers to obtain better water soluble, biodegradable, conductive materials [26]. It was found that these modified polyaniline materials have better cytocompatibility than traditional polyaniline.

**Scheme 1:**
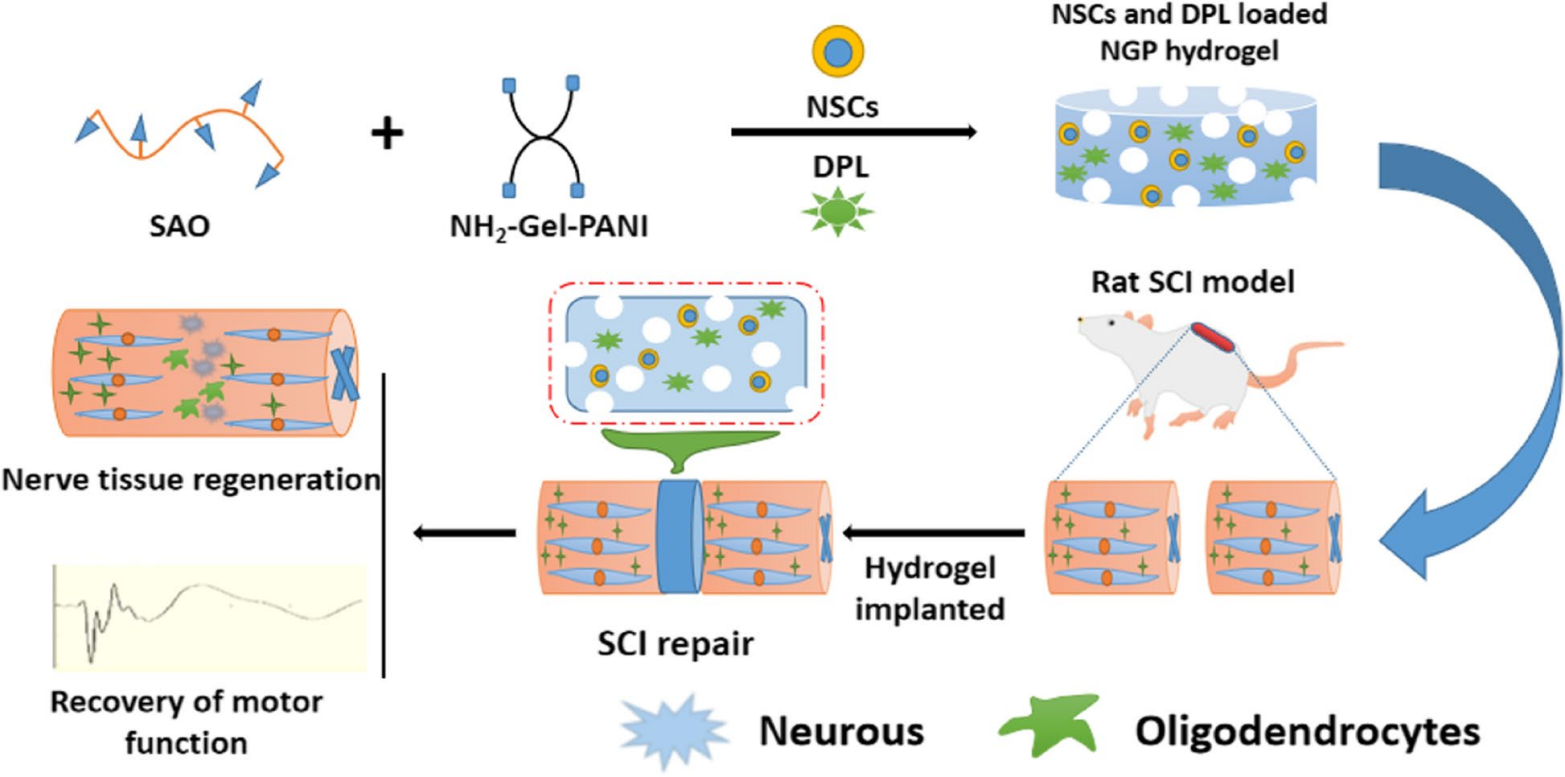
A brief schematic drawing of the application of NSCs and DPL-loaded NGP hydrogels for SCI repair

In this study, we used sodium hyaluronate oxide (SAO) and gelatine-g-polyaniline (NH_2_-Gel-PANI) as basic materials to construct a new injectable conductive hydrogel (NGP). A new conductive polymer NH2-Gel-PANI was prepared by grafting polyaniline onto the main chain of amino gelatin, on the basis of ensuring the electrical conductivity of the material, the biological toxicity of the material is reduced. Furthermore, gelatin components can simulate the chemical composition of natural extracellular matrix (ECM) and natural cell-binding motifs (such as RGD), which is conducive to cell adhesion and growth. Secondly, some experiments only use hydrogel materials to carry stem cells for stem cell therapy, but there are still defects of low cell survival rate and cells can not differentiate effectively, so this treatment method is not efficient. On the basis of carrying stem cells, we loaded DPL into the hydrogel by using the good drug-carrying properties of the hydrogel, and designed a drug combined with stem cells method for spinal cord injury treatment. This method can not only exert the drug effect in the damaged area, but also create a more ideal environment for cell growth and differentiation in the hydrogel and enhance the efficiency of stem cell therapy. As a treatment drug for Alzheimer’s disease, Donepezil (DPL) has been proved to have some effect on nerve damage in recent years, but there are few related studies. This study applied DPL to the treatment of spinal cord injury for the first time, further validates the drug efficacy of DPL and expands the application scope of this drug. NGP hydrogel was used to simultaneously deliver DPL and neural stem cells for the regeneration of orthotopic nerve tissue in a complete crosscut SCI model in rats (Scheme 1). HE staining, solid blue staining, immunofluorescence staining and BBB scoring were used to verify the effectiveness of this conductive hydrogel + DPL + NSC treatment system, which provides a new idea for the treatment of spinal cord injury.

## Materials and methods

### Materials

Type A gelatine was purchased from Sigma‒Aldrich (USA). Aniline, (3-(dimethylamino)propyl)-N′-ethylcarbodiimide hydrochloride (EDC), sodium periodate purchased from Aladdin Co., Ltd, China. Sodium hyaluronate was purchased from Shanghai Yuanye Biotechnology Co., Ltd, China. Ethylene glycol and sodium dihydrogen phosphate were purchased from Beijing Chemical Co., Ltd, China. The CCK-8 kit and live-dead cell staining kit were purchased from Dalian Meilun Biotechnology Co., Ltd, China. 4,6-Diamino-2-phenylindole (DAPI) and rhodamine-phalloidin were purchased from Sigma‒Aldrich (USA). The reagents for cell experiments were purchased from Abcam (UK). Haematoxylin and eosin (H&E) staining kits were purchased from Shanghai Biyuntian Experimental Reagent Co., Ltd, China. Fast blue staining kits were purchased from Solarbio Co., Ltd, China.

### Preparation of NGP electroactive hydrogel

Five grams of gelatine was dissolved in 0.1 M phosphate solution, and then 14 mL ethylenediamine and 5.35 g EDC were added to the solution. The above solution was diluted to 250 mL with a phosphate solution and then reacted in a water bath at 50 °C for 4 h. Subsequently, the obtained solution was put into a dialysis bag (Mw = 3500), and dialysis was carried out with deionized water for 3 days. Finally, the solution was freeze-dried to obtain white amino-gelatine (NH_2_-gelatine). Gelatine-g-polyaniline (NH_2_-Gel-PANI) was synthesized by a referenced method with modification. First, 14 mL of 15% NH2-gelatine solution, 162.88 mL of deionized water, 4.88 mL of CSA solution (50 mg/mL), and 244.32 mL of aniline solution were thoroughly mixed. After the above solution was completely cooled, 5.98 mL APS solution (100 mg/mL) was added to the solution and stirred at room temperature for 12 h. The black‒brown solution was slowly added to 600 mL acetone, and the flocculent precipitate was filtered and then washed with ethanol, n-methylpyrrolidone and ethanol twice. Finally, the final product (NH_2_-Gel-PANI) was dried in an oven for use. Stable injectable conductive hydrogels (NGPs) were synthesized by mixing SAO solution (10%, 200 µL) with NH_2_-Gel-PANI solution (20%, 0.1 M NaB_4_O_7_, 300 µL) uniformly and letting it stand for 30 s.

### Electroconductive hydrogel characterization

The sol-gel transition state of the hydrogels was observed by the vial tilting method. The internal structure of the hydrogel was observed by scanning electron microscopy (SEM, Zeiss EVO 18, Germany). The conductivity of the hydrogel was measured by cyclic voltammetry and an electrochemical workstation. A Fourier transform infrared (FTIR) spectrometer (Brucker, Germany) was used to detect the chemical composition of the hydrogels. The self-healing properties of the hydrogel were observed by cutting the hydrogel with a knife and reconnecting the broken surface. The biocompatibility of the hydrogels was evaluated by observing the changes in subcutaneous hydrogels and skin tissue.

### Loading and release of DPL in electroconductive hydrogels

In this study, the drug molecule DPL was loaded into the hydrogel by a physical adsorption method. The hydrogels are first immersed in the DPL solution (20 µM.) for 48 h. Subsequently, the hydrogels were removed, and the absorbance of the remaining DPL solution was measured with a UV‒visible spectrophotometer (SHIMADZU, Japan). The concentration of the remaining drug in the solution was calculated according to the standard curve method to calculate the drug loading of the hydrogel (M). After the hydrogels had absorbed DPL, they were immersed in 5 mL of PBS, which was stirred continuously at room temperature for 8 days in a rotary mixer. At each time point, the content of drug molecules in the solution was measured by UV‒visible spectrophotometry, i.e., the cumulative release (mR, mg/g). The cumulative release (R) rate was calculated according to the following formula: R (%)=(mR/m)×100%.

### Neural stem cell culture and identification

Embryos from a pregnant SD rat were collected at a gestational age of 16 days. NSCs were isolated from the cerebral cortex of foetal rats as previously described [27]. Neural stem cells were cultured and purified in a T25 flask (Corning, NY, USA) at a density of 50,000 MNS/cm^− 2^ and cultured in a 5% CO_2_ humidified incubator at 37 °C. The medium was replaced every two days, NSCs were passaged every 5 days, and cells between passages 2 and 6 were used for experiments. Subsequently, Nestin fluorescence staining was used to identify the NSCs. Briefly, the cells were fixed with 4% paraformaldehyde, and then 10% goat serum was added to seal the cells for 30 min. Subsequently, Nestin primary antibody was added to the cells and refrigerated overnight at 4 °C. After the cells were washed with PBS, Cy3 fluorescent secondary antibody and DAPI were added for staining. Finally, the NSCs were observed by fluorescence microscopy (Olympus, Japan).

### Cell differentiation during proliferation

To observe whether NGP hydrogels are cytotoxic, the NGP hydrogel was evenly applied on the surface of the slide. After the hydrogel formed a complete gel, it was placed in a culture of neural stem cells. Then, the cells were placed in a cell culture incubator containing 5% CO_2_ at 37 °C and cultured with proliferation medium. At 1, 3 and 5 days of cell culture, cells were stained with Calcein AM and PI and observed under a fluorescence microscope to evaluate the cytotoxicity of the NGP hydrogels. To detect the effects of NGP hydrogels and DPL on the proliferation of neural stem cells, the CCK-8 method was used to detect cell proliferation in different experimental groups. In brief, there were 4 experimental groups: the control group, the pure NGP hydrogel group, the pure DPL group (8 µg/mL DPL added to the cell medium), and the NGP + DPL group. After all samples were exposed to UV for 2 h, 2 × 10^4^ NSCs were inoculated in each experimental group. After NSCs were cultured for 1, 4 and 7 days, the medium was removed, and 100 µl CCK-8 solution was added and cultured at 37 °C for 4 h. Finally, 100 µL dissolved liquid was transferred to a new 96-well plate, and the absorbance value at 450 nm was determined by a multifunctional microplate scanner (Thermo, USA).

### Cell differentiation within hydrogels

To detect the effects of NGP hydrogels and DPL on the differentiation of neural stem cells, these cells were cultured in proliferation medium for 2 days, and the medium was replaced with NSC differentiation medium on the 3rd day. The cell differentiation assay included immunofluorescence staining and real-time PCR, which was performed after cells were cultured for 7 days. NSCs cultured under the various conditions for 7 days were collected, and total RNA was isolated. Then, cDNA was synthesized using a PrimeScript RT reagent kit (Takara Bio, Japan) according to the manufacturer’s instructions. The primer sequences specific for the target gene used for qRT‒PCR are listed in Table 1. The qRT‒PCR procedure started at 95 °C for 10 min, followed by 40 cycles at 95 °C for 30 s, 58 °C for 1 min and 72 °C for 1 min. GAPDH was used as an internal reference gene.

**Table 1:** List of Genes and Primer Nucleotide Sequences

Gene	Forward primer sequence	Reverse primer sequence
**Tuj-1**	5-GATCGGAGCCAAGTTCTG-3'	5-GTCCATCGTCCCAGGTTC − 3'
**GFAP**	5- GCAGACCTTCTCCAACCTG-3'	5- ACTCCTTAATGACCTCTCCATC-3'
**OSP**	5- CGACGCCAAAGAGGAACAG-3'	5- GCCAAGTTCAGGTCCTGCAT-3'
**GAPDH**	5-TCGCCAGCCGAGCCA − 3'	5- CCTTGACGGTGCCATGGAAT − 3'

To further detect the effect of different composite scaffolds on cell osteogenic differentiation, NSCs in different groups were stained by immunofluorescence. The cells were first fixed with 4% paraformaldehyde and then treated with 1% Triton X-100. The cells were sealed with 10% goat serum, and diluted Tuj-1, GFAP and OSP primary antibodies were then added to the cells at 4 °C overnight. After the cells were washed with PBS, Cy3 fluorescent secondary antibody and DAPI were added to the cells. Finally, the cells were observed under a fluorescence microscope.

### Rat spinal cord injury repair

Female Sprague Dawley (SD) rats were randomly allocated to four groups (control group, NGP hydrogel group, NGP + NSC group and NGP + DPL + NSC group) and were used for the spinal cord injury repair of hydrogels. All experimental animals were provided by Liaoning Changsheng Biotechnology Company and kept in the Animal Room of Public Health College of Jilin University. In brief, SD rats were anaesthetized and fixed on the bench, and then the dorsal thoracic vertebrae of the rats were disinfected. The paravertebral muscles were separated, and the exposed vertebral plates were removed using a rongeur. After exposing T9/T10 spinal cord tissue, scissors were used to cut 2-mm-long sections of spinal cord tissue. Blank treatment or injection of corresponding hydrogel scaffolds. Within 7 days after surgery, penicillin was injected intramuscularly into all experimental animals, and the animals’ defecation, mental state, wound infection and other conditions were observed every day.

### Detection of motor and electrophysiological function in rats

On postoperative weeks 1–6, the effect of different hydrogels on rat motor function recovery was examined with the BBB (Basso–Beattie–Bresnahan) score and evaluated once a week. In this experiment, the recovery of motor evoked potentials (MEPs) in experimental rats was detected by electromyography. In short, the stimulation electrodes were inserted into the motor area of the cerebral cortex and the contralateral gastrocnemius muscle of the rats, a 140 V stimulation intensity was given for stimulation, and the changes in MEPs on electromyography were observed.

### Histological evaluation

On postoperative week 6, spinal cord tissue samples were removed and fixed with paraformaldehyde. Then, spinal cord samples were placed in paraffin wax and cut into tissue slices. Subsequently, tissue slices were stained with Luxol Fast Blue (LFB), H&E, Tuj-1, NF200 and CS56 in accordance with standard protocols, and samples were observed using a light microscope and fluorescence microscope.

### Statistical analysis

Statistical analysis was performed using one-way analysis of variance to determine significant differences. When *p* < 0.05 (*), the data were considered statistically significant. The data are shown as the mean ± standard deviation (SD).

## Results and discussion

### Characterization of the NGP hydrogels

**Fig. 1:**
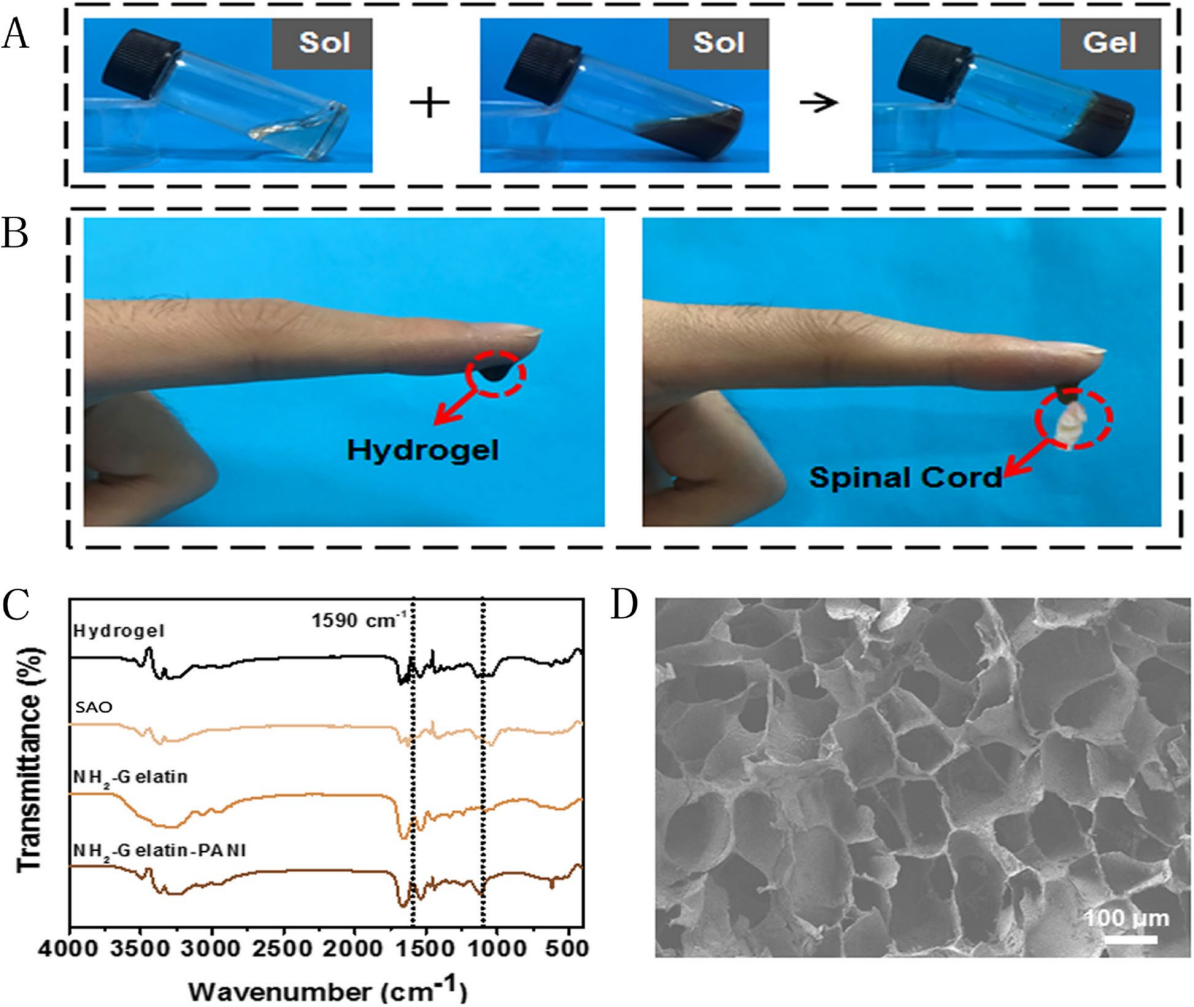
**A** Images of the SAO and NGP solutions and the NGP hydrogels. **B** Adhesion of NGP hydrogels to spinal cord tissue. (**A**) FITR spectra of NH_2_-Gelatin, NH2-Gelatin-PANI, SAO and NGP hydrogels. **D** SEM images of NGP hydrogels, scale bar lengths are 100 μm

The NGP hydrogel in this experiment was synthesized by a two-step reaction. First, polyaniline was grafted onto the amino-gelatine backbone to improve the biocompatibility and degradability of the conductive polymer. Finally, NH_2_-Gel-PANI copolymer and SAO were mixed, and NGP hydrogels were prepared through dynamic imide bonds formed by amino groups and aldehyde groups. As shown in Fig. 1A, SAO solutions were viscous liquids. However, upon mixing the NH_2_-Gel-PANI copolymer and SAO, a solid gel formed in a few minutes. The NGP hydrogels were soft and could stably hang on the bottom of an inverted glass vial, indicating their solid-like properties. Furthermore, as shown in Fig. 1B, the NGP hydrogels had good adhesion to fresh spinal cord tissue. The good adhesion of hydrogels can make the material more closely connected with the damaged spinal cord, which is conducive to the growth and repair of nervous tissue. Subsequently, the microstructure of the NGP hydrogels was observed by SEM. As shown in Fig. 1D, the inside of the NGP hydrogel presents an obvious porous structure, which is conducive to the transport of nutrients and the removal of metabolic waste. More importantly, the pore size of NGP hydrogels is approximately 100 μm, which matches the size of the nerve ball formed by the NSCs, providing a strong guarantee for carrying NSCs. We then studied the chemical composition of NH_2_-gelatine, NH_2_-gelatine-PANI, SAO and NGP hydrogels by Fourier infrared spectroscopy (FTIR). As shown in Fig. 1C, the FTIR spectrum of NH_2_-gelatine exhibited three characteristic peaks at 1631 cm^− 1^, 1523 cm^− 1^, and 1236 cm^− 1^, which represented the characteristic peaks of amide I, II, and III, respectively. The band at 3279 cm^− 1^ represents the stretching vibrations of overlapping N-H and O-H. The FITR spectrum of the NH_2_-gelatine-PANI copolymer exhibited not only the characteristic peaks of NH_2_-gelatine at 1631 cm^− 1^ but also the characteristic peaks of polyaniline at 1564 cm^− 1^ and 1488 cm^− 1^, indicating that polyaniline was successfully grafted to the main chain of amino-gelatine. The FITR spectrum of NGP hydrogels shows the overlap of SAO and NH2-gelatine-PANI characteristic peaks, indicating that the NGP hydrogels are composed of SAO and NH2-gelatine-PANI.

**Fig. 2:**
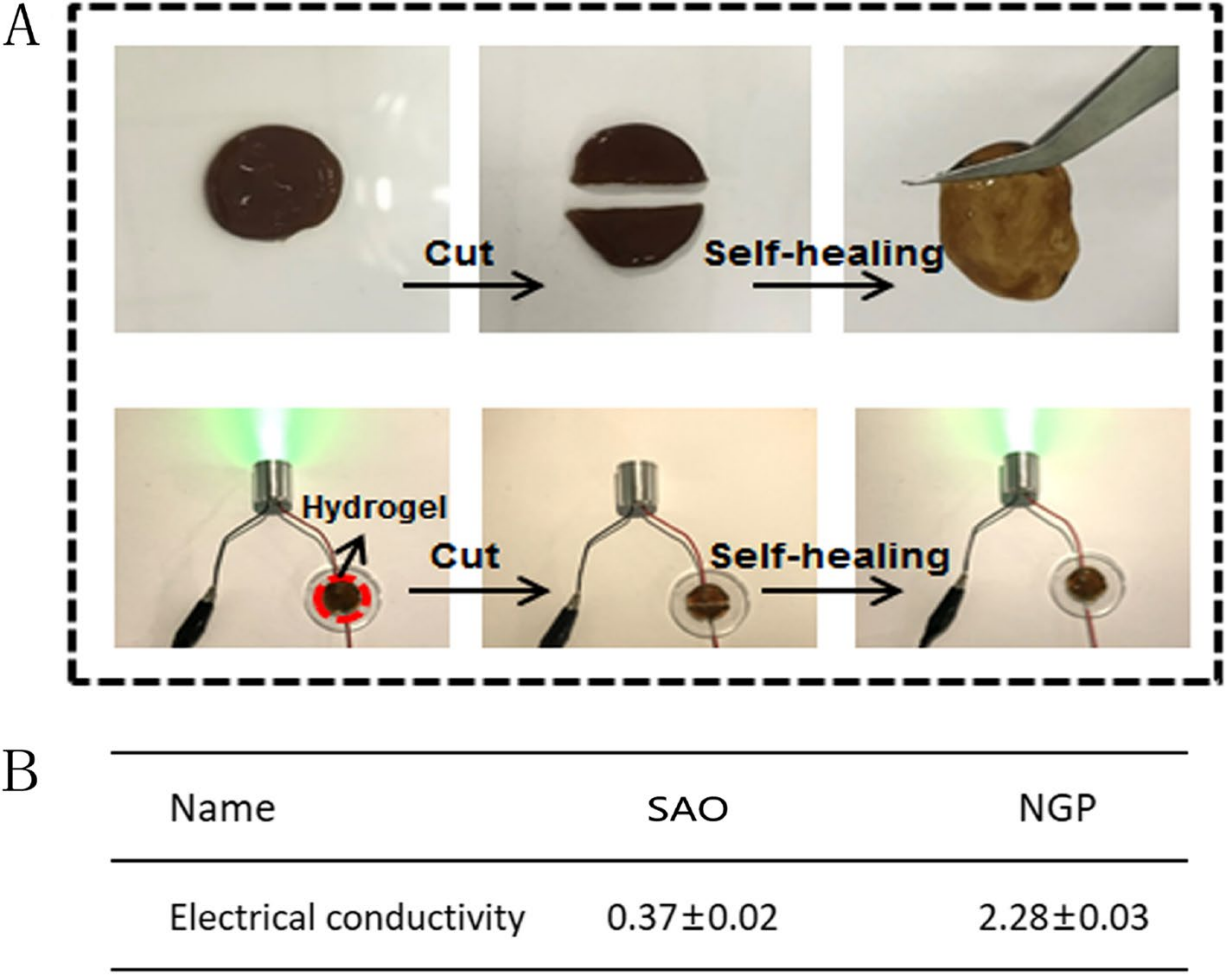
**A** The display of the self-healing ability of NGP hydrogels. **B** The electrical conductivity results of NGP hydrogels

Many studies have demonstrated that electroactive materials are more conducive to information exchange between nerve cells, which can better promote the growth of nerve cells. As shown in Fig. 2B, the electrical conductivity of the material increased from 0.37 S/m to 2.28 S/m with the addition of NH_2_-gelatine-PANI. The conductivity value of NGP hydrogels is in the range of 1–10 S/m, which is the optimal range for the growth of nervous tissue [28]. Macroscopic self-healing tests and electric circuit recovery experiments were performed to investigate the self-healing ability of the NGP hydrogels. As shown in Fig. 2A, the round sheet NGP hydrogel was first cut into two segments, and then the two segments were put together again and placed at room temperature for 10 min. Subsequently, it was found that the hydrogel healed again as a whole, without breaking under gravity. More importantly, the conductivity of the NGP hydrogel was also well restored, as the brightness of the green bulb barely changed before and after the hydrogel healed. According to the above results, NGP hydrogels have excellent electrical conductivity, self-healing, and adhesion properties, which are essential for tissue engineering applications, as NGP hydrogels can tightly attach to the surface of the damaged spinal cord tissue and re-establish electrical conduction to the damaged tissue.

**Fig. 3:**
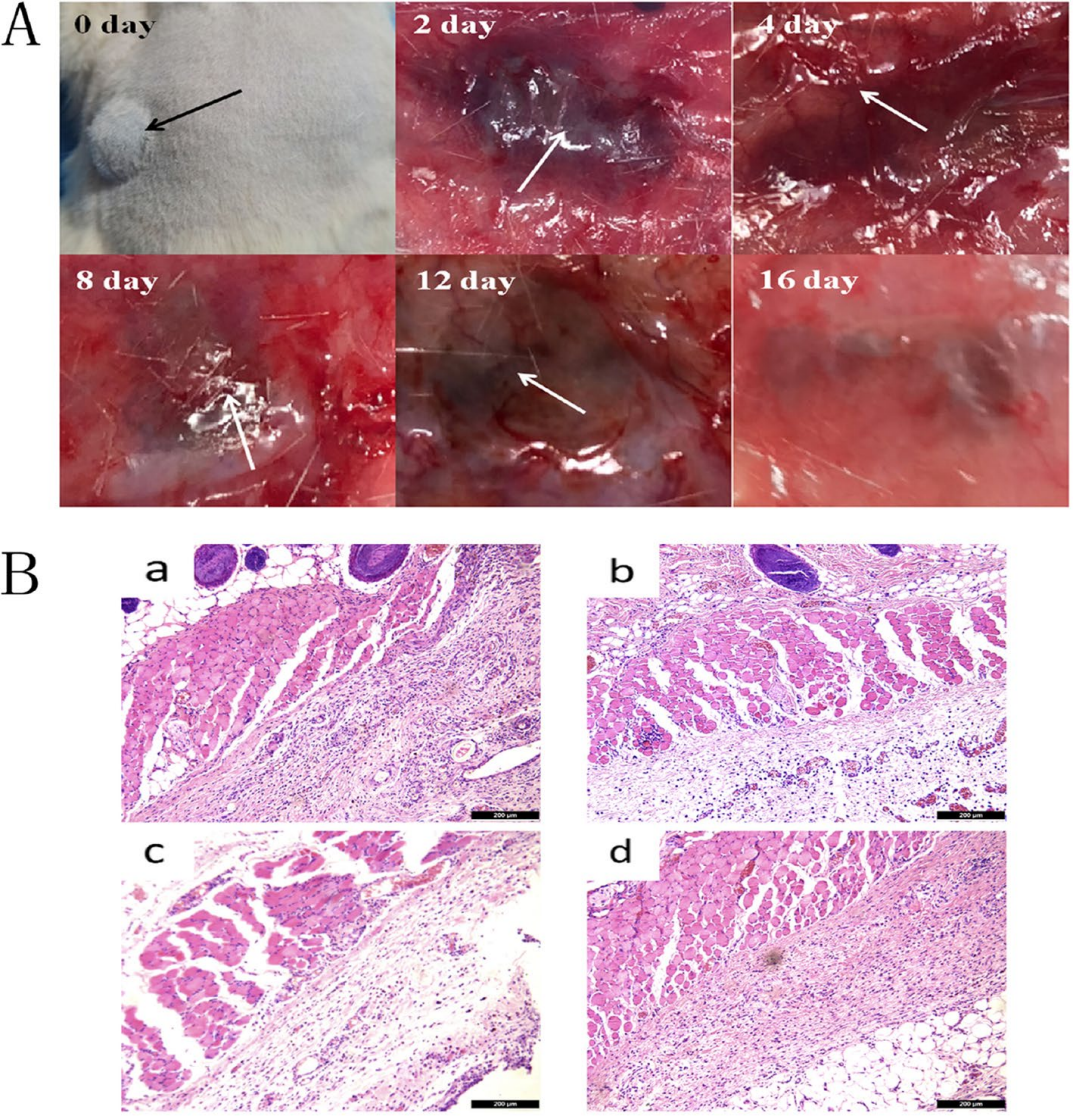
**A** In vivo degradation images of NGP hydrogels at different time points. **B** Effect of NGP hydrogel on surrounding skin tissue after subcutaneous implantation, a: Normal mice b: 4 days c: 8 days d: 16 days, scale bar lengths are 200 μm

As an implantable material, NGP hydrogels need to have the necessary degradability and biocompatibility. Therefore, we implanted the NGP hydrogel into the skin of rats and evaluated its biocompatibility by observing changes in the hydrogel and subcutaneous tissue. As shown in Fig. 3A, the residual amount of hydrogel under the skin gradually decreased over time, with only a small amount remaining after 16 days. HE staining was used to evaluate the skin histopathological changes after the hydrogel was implanted subcutaneously. As shown in Fig. 3B, there was inflammatory cell infiltration in the subcutaneous tissue with a certain degree of inflammation on the 4th to 8th day after hydrogel injection. After 16 days of hydrogel implantation, the skin tissue basically returned to a normal state. The above results demonstrated that NGP hydrogels can be fully absorbed by the body without causing a particularly severe immune response.

**Fig. 4:**
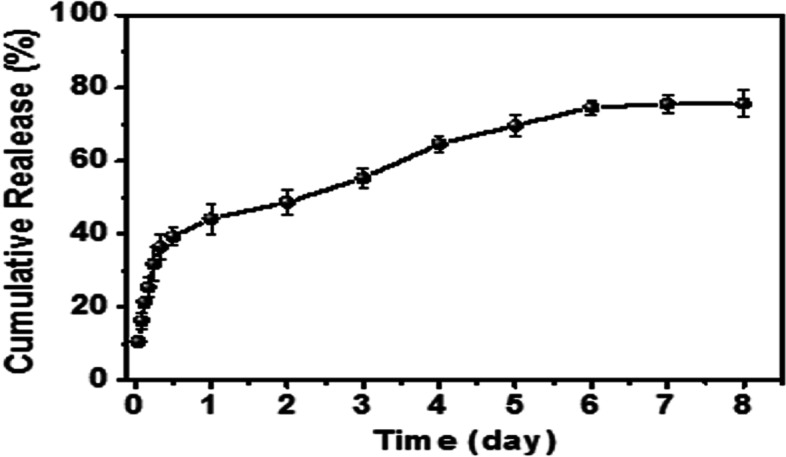
In vitro drug (DPL) release of the NGP hydrogel over 8 days; error bars represent the standard deviation for *n* = 3

In this study, one of the main functions of NGP hydrogels was to deliver DPL. Drug release is closely related to the properties of the hydrogels, the interaction force between the hydrogels and drug molecules, and the properties of the drug. As shown in Fig. S1 A and B, the ultraviolet spectrum results showed that DPL had a maximum absorption peak at 313 nm. Therefore, the absorption peak of known concentration DPL at 313 nm was used to draw the drug standard curve. An in vitro release assay was conducted with the solution soaking method to evaluate DPL release rule from the NGP hydrogel. As shown in Fig. 4, after an 8-day release experiment, we tested the sustained release of DPL. The results showed that the NGP hydrogel can control the slow release of the drug, with a cumulative release rate of 75% by the eighth day. The sustained drug release behaviour of NGP hydrogels may be due to the dense intermicellar structure formed by NH_2_-gelatine-PANI and SAO. At the same time, the highly packed supramolecular structure can reduce the diffusion coefficient inside the hydrogel, resulting in sustained drug release [29]. Based on these results, slow and sustained drug release can be achieved using the NGP hydrogel system, which can reduce the frequency of administration and increase patient compliance.

### Influence of NGP and DPL on NSC proliferation

**Fig. 5:**
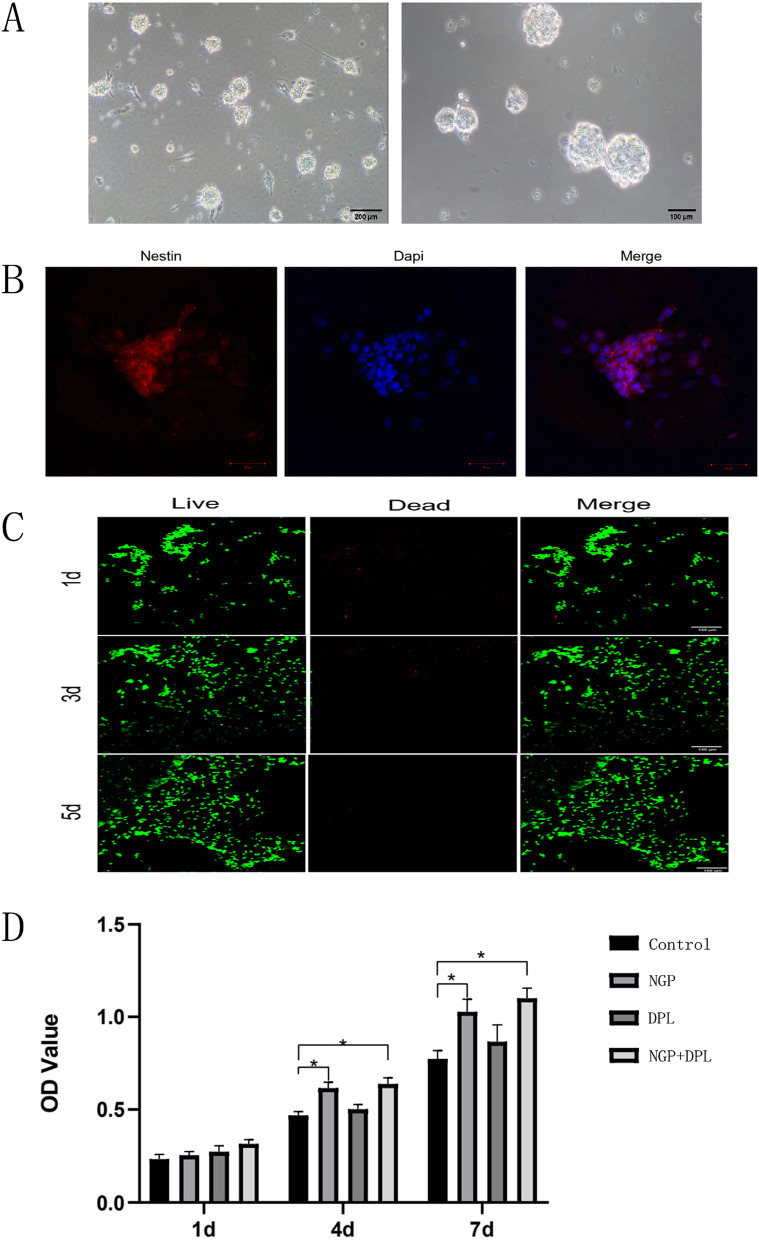
**A** Morphology of NSCs under a light microscope. Left: ruler: 200 μm; right: ruler: 100 μm. **B** Nestin immunofluorescence identification of NSCs. **C** Live and dead cell staining of NSCs on NGP hydrogels; scale bar lengths are 100 μm. **D** Cell proliferation of NSCs cultured in the control, NGP, DPL and NGP + DPL groups, *P < 0.05, error bars represent the standard deviation for *n* = 3

In this study, the extraction and culture of NSCs was one of the most important steps. While NSCs were used to evaluate the biological effects of NGP hydrogels and DPL, NSCs were also required to be transplanted into the injured spinal cord as seed cells. NSCs were obtained by isolating the cerebral cortex tissue of foetal mice. As shown in Fig. 5A, NSCs were grown in suspension and formed nerve spheres with a diameter of approximately 100 μm. Subsequently, Nestin immunofluorescence staining was used to identify the extracted NSCs. As shown in Fig. 5B, the cells showed a positive immune response to nestin under the microscope, and the positive rate of nestin basically reached 100%, which demonstrated that the extracted cells were NSCs. To further verify the biocompatibility of NGP hydrogels, NSCs were implanted on the surface of the hydrogel and stained with calcein (live cells) and PI (dead cells). As shown in Fig. 5C, after 1, 3 and 5 days of cell culture, a large number of living cells and a small number of dead cells were observed on the NGP hydrogel, which shows that the NGP hydrogel has good cytocompatibility. Subsequently, to further verify the effects of hydrogels and DPL on the proliferation of NSCs, we seeded NSCs in the control, NGP hydrogel, pure DPL and NGP hydrogel + DPL experimental groups. Figure 5D shows the proliferation of NSCs in different groups. After 1 day of culture, there was no significant change in cell proliferation among the groups. On the 3rd day, the proliferation rate of cells in the NGP and NGP + DPL groups was significantly higher than that in the control and DPL groups (*P* < 0.05), demonstrating that the NGP hydrogels can effectively improve cell proliferation. After 7 days of cell culture, cell proliferation in each group was similar to that on the 3rd day, but cell proliferation in the DPL group improved to a particular extent, and the cell proliferation rate was higher than that in the control group. The results of the above cell proliferation experiments showed that both NGP hydrogels and DPL could promote the proliferation of NSCs to some extent, but the effect of NGP hydrogels was more obvious.

### Influence of NGP and DPL on NSC differentiation

**Fig. 6:**
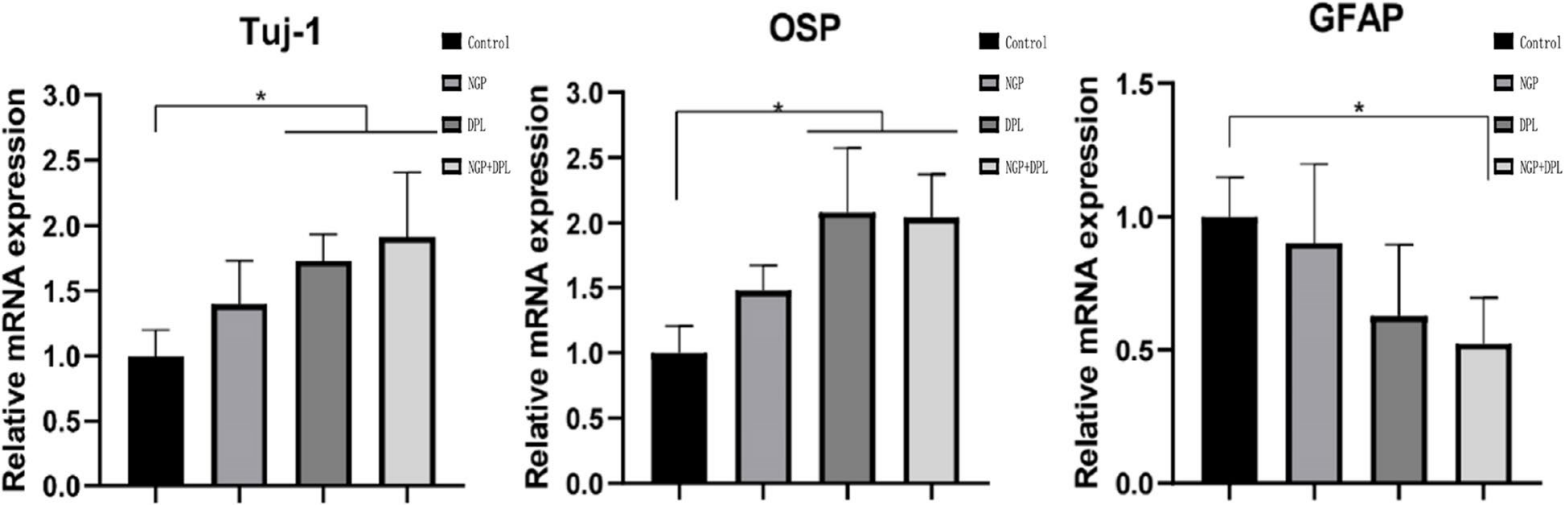
Quantitative real-time PCR analysis of Tuj-1, OSP and GFAP expression in NSCs seeded in different groups. *P < 0.05, error bars represent standard deviation for *n* = 4

Nervous tissue regeneration involves processes such as cell proliferation, differentiation and neural pathway reconstruction, which are regulated by various genes. For example, the upregulated expression of Tuj-1 gene expression represents the production of new neurons, and the upregulated expression of OSP and GFAP genes often represents the presence of a large number of oligodendrocytes and astrocytes [30, 31]. Therefore, in this study, the Tuj-1, OSP and GFAP genes were selected to detect the differentiation of NSCs by RT‒PCR. As shown in Fig. 6, the Tuj-1 gene expression results showed that after 7 days of culture, the mRNA expression levels of Tuj-1 in the NGP, DPL and NGP + DPL groups were significantly higher than those in the control group, indicating that the NGP hydrogel and DPL could increase the neuronal differentiation of NSCs. The trend of OSP gene expression was similar to that of Tuj-1. Furthermore, the mRNA expression levels of Tuj-1 and OSP were significantly higher in the DPL group than in the NGP group, indicating that the effect of DPL in promoting neural differentiation was more obvious than that of the NGP hydrogel. The expression of the GFAP gene showed a downward trend in the control, NGP, DPL and NGP + DPL groups. Both NGP and DPL significantly reduced the expression of GFAP, and the effect of DPL was more significant. The inhibitory effect was most obvious when DPL and NGP were used in combination.

**Fig. 7:**
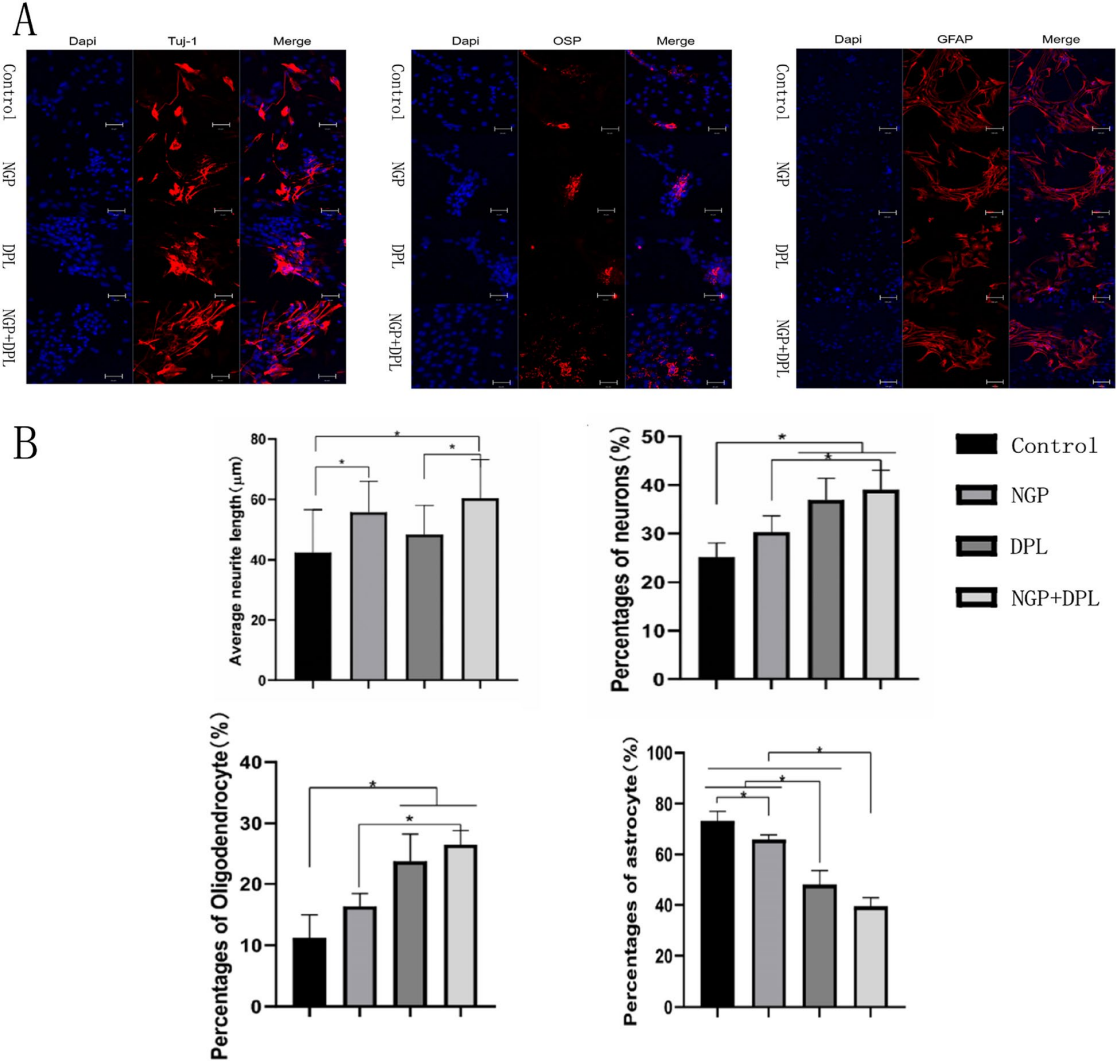
**A** Immunofluorescent images for Tuj-1, OSP and GFAP expression by NSCs seeded on different groups. DAPI staining for nuclei (blue) and Cy3-conjugated secondary antibody for protein (red), scale bar lengths are 50 μm. **B** Axon length of new neurons and the proportion of new neurons, oligodendrocytes and astrocytes in each group, *P* < 0.05, error bars represent standard deviation for *n* = 3

To further examine the differentiation ability of NSCs in different groups, immunofluorescence staining was used to investigate the differentiation of NSCs. After incubation, cells that were mostly positive for GFAP with little positivity for Tuj-1 and OSP were observed in the control group (Fig. 7A). In the DPL group, increased Tuj-1 and OSP expression was observed in the fluorescence images of the samples, significantly exceeding that in the NGP groups. When DPL and NGP were combined, Tuj-1 and OSP expression was further increased in the samples. In terms of GFAP protein expression, we found that protein expression was lower in the DPL group than in the NGP group. The expression analysis of proteins and genes related to NSC differentiation showed a high degree of agreement between the two. Furthermore, the neurite length of newborn neurons in both groups treated with NGP hydrogel was significantly greater than that in the other groups, while no such phenomenon was observed in the DPL group (Fig. 7B). The above results indicate that our conductive hydrogel itself can promote the growth and extension of newborn neurons, which is the ability that DPL does not have. In conclusion, DPL can promote the differentiation of NSCs into new neurons and oligodendrocytes, but it cannot promote the growth and extension of new neurons like NGP hydrogels, and the combined application of NGP and DPL can achieve complementary advantages.

### NSC- and DPL-loaded NGP hydrogel for spinal functional recovery

**Fig. 8:**
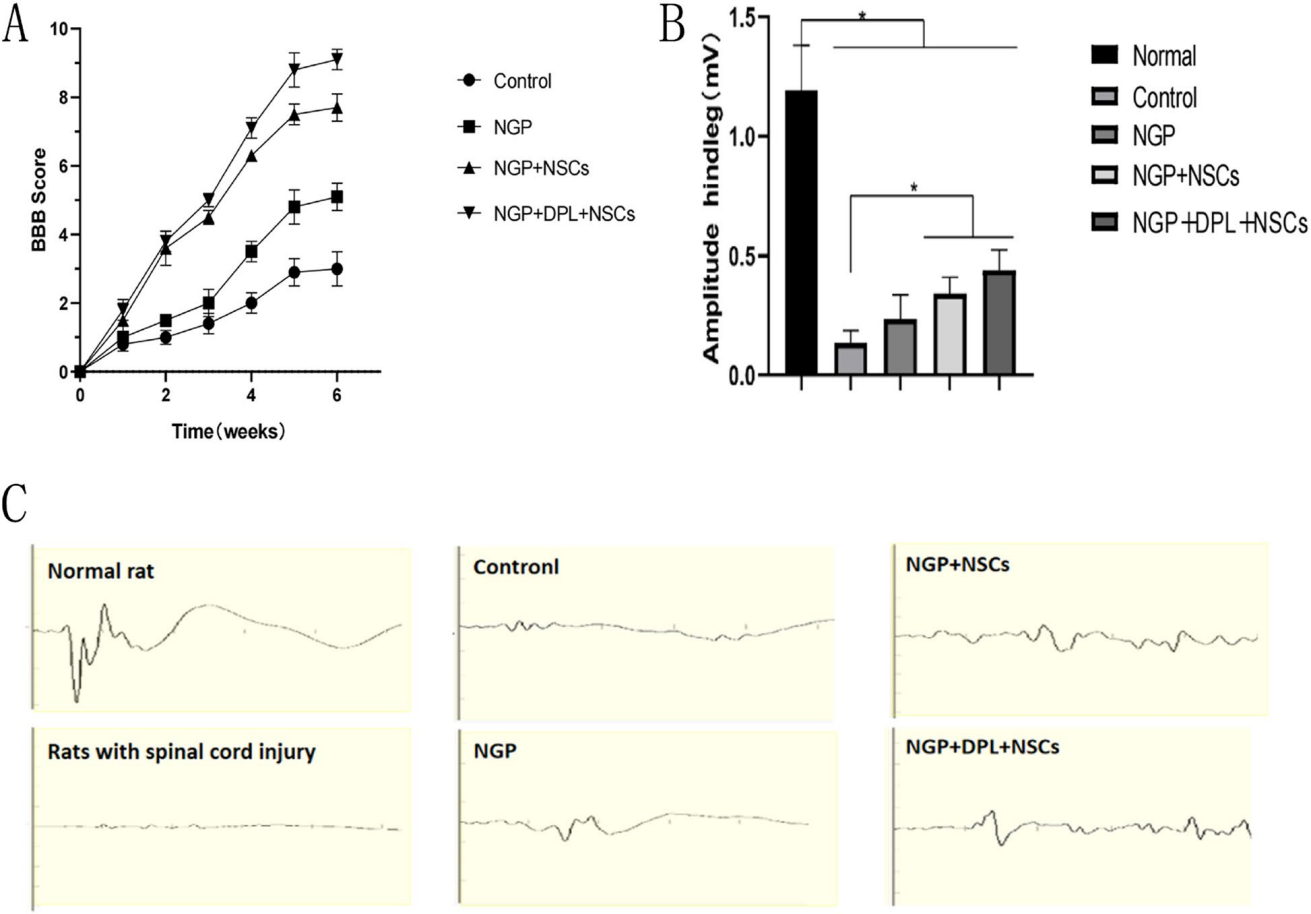
**A** BBB scores of different treatment groups at ten weeks postinjury. **B** The results of MEP amplitude measurement in each group. **C** Electromyography of rats in each experimental group. *P < 0.05, error bars represent standard deviation for *n* = 6

After confirming that the NGP hydrogel and DPL could collectively improve the proliferation and neuronal differentiation and restrict astrocytic differentiation of NSCs in vitro, we further decided to use NGP hydrogels to deliver both DPL and NSCs, transplant NSC/DPL-loaded NGP hydrogel into a rat model of spinal cord injury and observe its therapeutic effect in vivo. First, locomotor recovery was assessed using the Basso–Beattie–Bresnahan locomotor rating scale (BBB scale) for six weeks after injury. As shown in Fig. 8A, one week after surgery, the motor function of the rats gradually recovered, and the BBB score of the NGP + NSC and NGP + NSC + DPL groups increased slightly over time, but there was no statistical significance between the four groups. After 2 weeks, the motor function score of the control group increased slowly, and the recovery of the NGP group was slightly better than that of the control group. Furthermore, compared with the NGP group, the NGP + NSC and NGP + DPL + NSC groups had higher BBB scores. After 6 weeks, the recovery of motor function in the NGP + DPL + NSC group was the best, the BBB score of the NGP + DPL + NSC group was close to 10 points, and the BBB score of the NGP + NSC group was approximately 8 points, which was higher than that of the control group and the NGP group. These results demonstrated that NSC- and DPL-loaded NGP hydrogel treatment exerted an optimal effect on locomotion recovery after implantation. To further observe the recovery of motor function in rats, the recovery of rat nerve electrophysiological signals was evaluated by an electromyography instrument. As shown in Fig. 8B, we first measured the motor evoked potential (MEP) of the rats before and immediately after surgery and found that the amplitude of MEP decreased from approximately 1.19 mV to 0, indicating that the spinal cord was completely severed. Six weeks after surgery, a gradual increasing trend of MEP amplitude in the four groups was found. The NGP + DPL + NSC group had the highest amplitude, while the control group had the lowest amplitude. The MEP amplitude was as follows: NGP + NSC + DPL group (0.44 mV) > NGP + NSC group (0.34 mV) > NGP group (0.26 mV) > Control group (0.12 mV). According to the above results, we found that the combination of NGP, DPL, and NSCs has a very good effect on the recovery of motor function and nerve conduction function in injured nerve tissue.

### Histologic analysis

**Fig. 9:**
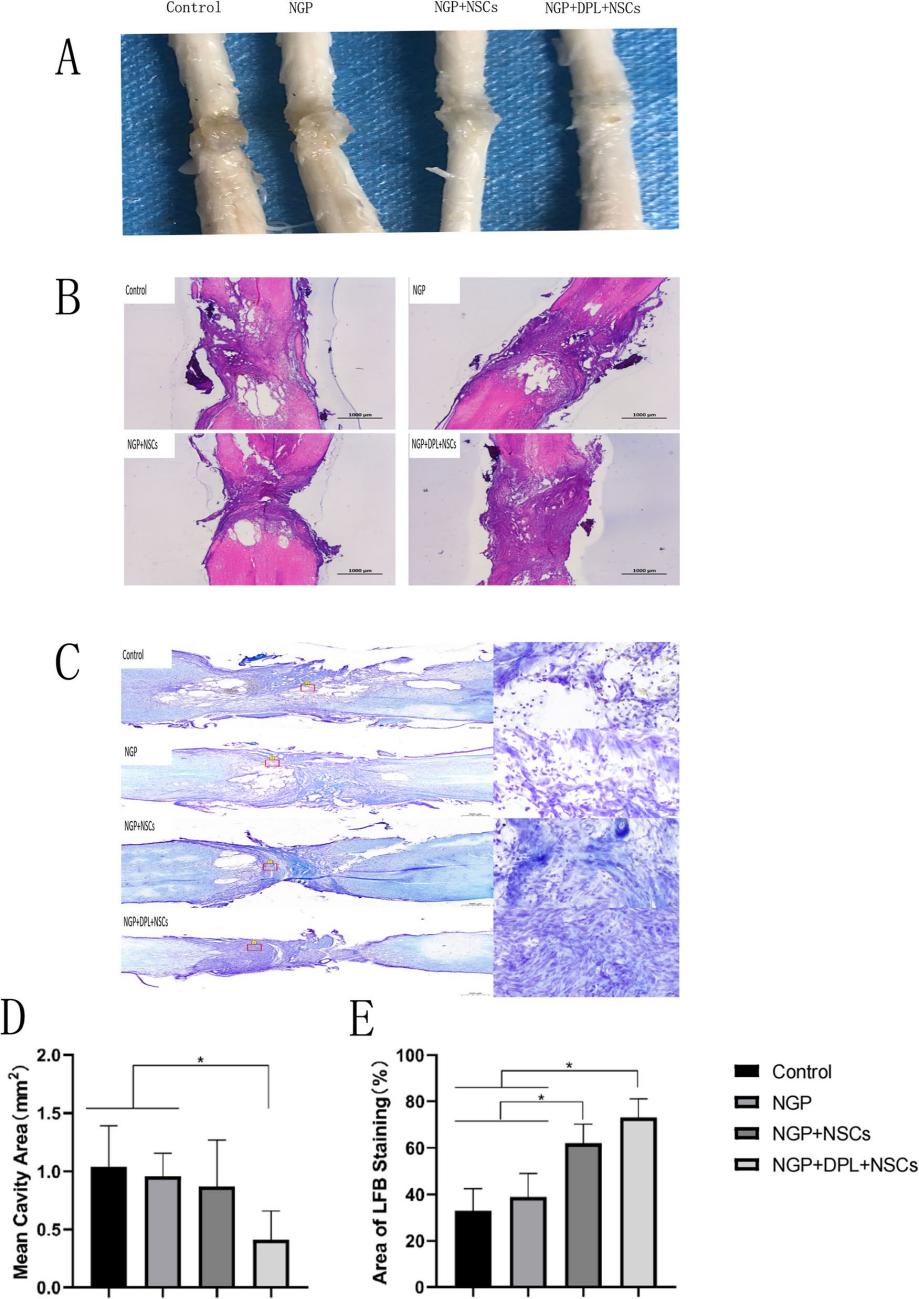
**A** Image of spinal tissue specimen. **B** H&E staining of spinal cord tissues in different groups; scale bar lengths are 1000 μm. **C** LFB staining of spinal cord tissue in different groups, Left: scale bars = 500 μm; right: scale bars = 50 μm. **D** Semiquantitative analysis of the cystic cavity area in different groups, *P < 0.05, error bars represent standard deviation for *n* = 3. **E** Semiquantitative analysis of the LFB staining area in different groups, *P < 0.05, error bars represent standard deviation for *n* = 3

Six weeks after surgery, the rats were killed, and spinal cord tissue was removed. We first observed the spinal cord tissue. As shown in Fig. 9A, the implanted hydrogels in the defect of the spinal cord basically degraded after being treated for 6 weeks. From the control group to the NGP + DPL + NSC group, the colour of the injured spinal cord tissue segments changed from dark to light, and the amount of scar tissue showed a gradual downward trend. Subsequently, HE staining was performed on the spinal cord tissues of rats. As shown in Fig. 9B, there were different degrees of tissue reconnection in the spinal cord injury segments. Notably, among the four groups, the NGP + NSC + DPL-treated group had the most complete tissue connections. Furthermore, more cystic cavities were observed in the control group than in any of the other groups, and the encapsulation of NSCs and DPL in the NGP hydrogel significantly reduced the cystic area (Fig. 9D).

LFB staining was used to observe the regeneration of the myelin sheath in the injury centre area, and semiquantitative analysis was performed on the myelin-positive area. As shown in Fig. 9E, the semiquantitative analysis showed that the mean value of myelin-positive areas in the NGP group was slightly higher than that in the control group, but there was no significant difference (P > 0.05). The myelin-positive areas in the NGP + NSC group were significantly larger than those in the NGP group (*P* < 0.05). In all groups, the myelin-positive areas in the NGP + DPL + NSCs group were significantly higher than those in the other three groups (*P* < 0.05). As shown in Fig. 9C, the myelin sheath was bright blue, and the control and NGP groups had lighter staining and less myelin sheath tissue, while the staining intensity and blue myelin sheath tissue density in the NGP + NSCs and NGP + DPL + NSCs groups significantly increased, indicating that the combination of DPL and NSCs can effectively promote myelin regeneration.

**Fig. 10:**
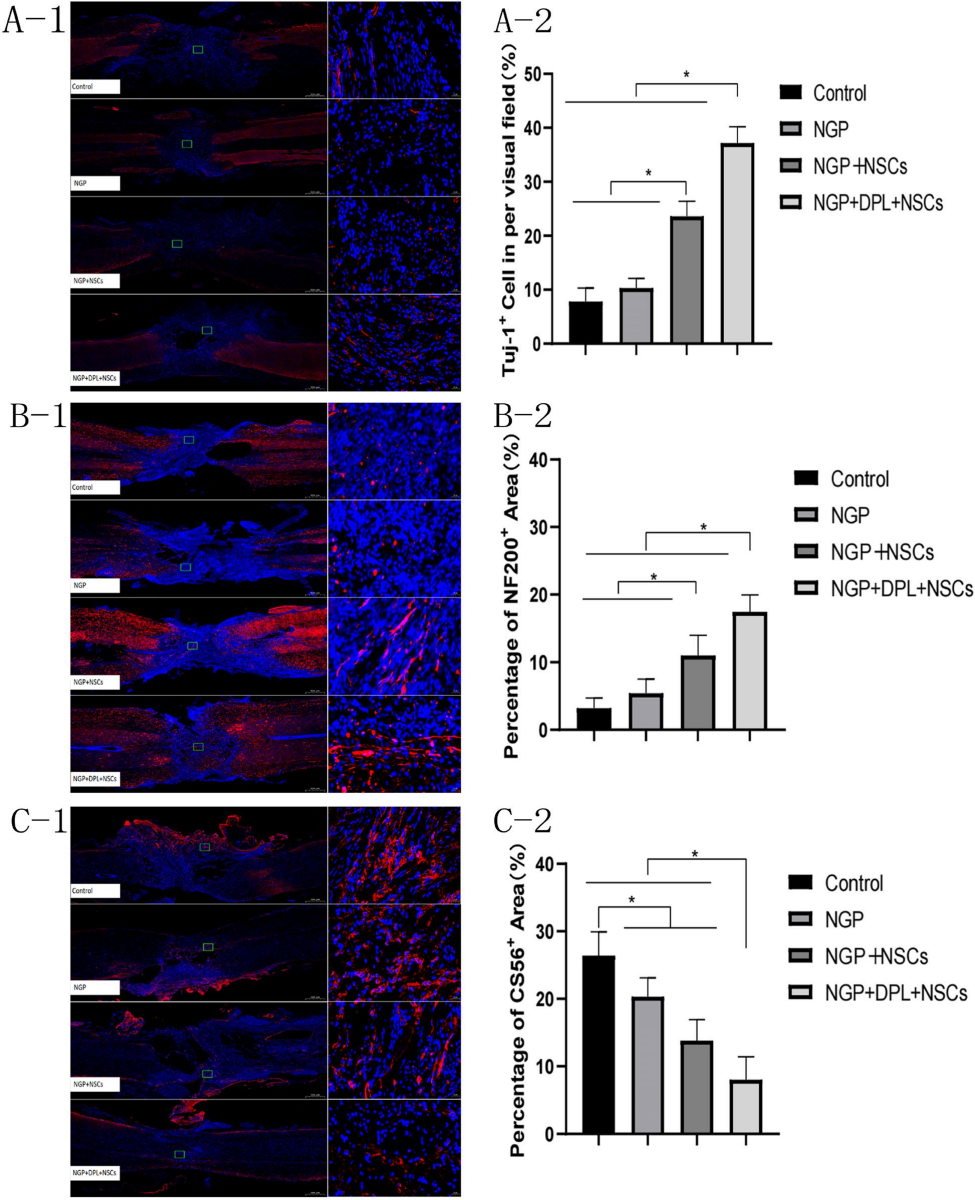
Immunofluorescent images for Tuj-1 (A-1), NF200 (B-1) and CS56 (C-1) at the lesion sites of the control group and NGP group, Left: scale bars = 500 μm. Right: scale bars = 20 μm. Percentage of Tuj-1 (A-2), NF200 (B-2) and CS56 (C-2) positive area in each group, *P < 0.05, error bars represent standard deviation for *n* = 3

Subsequently, immunofluorescence staining was used to investigate the formation of newborn neurons (Tuj-1 positive), axons (NF200 positive) and glial scars (CS56 positive) in the injured area of spinal cord tissue. As shown in Fig. 10. A and B, few Tuj-1- and NF200-positive neurons were located at the lesion sites of the control group and NGP group, and the number of Tuj-1- and NF200-positive neurons was significantly lower than the number of GFP-positive neurons. Compared with the control and NGP groups, more Tuj-1- and NF200-positive neurons were distributed at the lesion sites of the NGP + NSC and NGP + DPL + NSC groups. Among all groups, the NGP + DPL + NSC group had the highest percentage of Tuj-1- and NF200-positive areas. The glial scar formed by astrocytes in the lesion area was labelled by CS56 immunostaining. As shown in Fig. 10C, CS56 was highly expressed in the control group. The percentage of positive CS56 area decreased in the NGP group compared with the control group (*P* < 0.05). CS56 expression declined more significantly in the NGP + NSC group and the NGP + DPL + NSC group (*P* < 0.05). Immunofluorescence staining of Tuj-1, NF200 and CS56 further confirmed that NSCs transplanted along with NGP hydrogel could successfully differentiate into neurons and generate axons for normal physiological activities, all of which played a positive role in spinal cord functional repair. The therapeutic effect can be further expanded by using DPL as an inducer of directed differentiation. At the same time, NGP hydrogels loaded with NSCs and DPL can minimize the formation of astrocytes, thus inhibiting the formation of glial scars and promoting nerve regeneration.

Stem cell and drug therapy are widely used in the treatment of spinal cord injury; however, there are always some problems in the application of these therapies. For example, many studies have shown that exogenous NSC transplantation has a limited effect on motor function recovery in animals with spinal cord injury [32]. This may be due to the low survival rate of NSCs and the high differentiation rate of astrocytes during transplantation [33]. Furthermore, many drugs fail to achieve the desired therapeutic effect because they cannot cross the blood‒spinal barrier. Even drugs that permeate the blood–brain barrier often require high systemic doses to achieve therapeutic levels at the injury site, resulting in deleterious side effects [8]. Therefore, the use of stem cells or drugs to treat spinal cord injury often requires an efficient carrier. In recent years, conductive hydrogels have received significant attention for the local delivery of cells and drugs. These hydrogels are highly hydrated and have mechanical strength similar to nerve tissue, making them excellent candidates for spinal cord nerve tissue scaffolds. More importantly, conductive hydrogels can mimic the high electrical conduction characteristics of the natural spinal cord to promote the functional recovery of interrupted conduction nerve pathways, maintain the endogenous conductive microenvironment, and promote the regeneration of spinal nerves. Therefore, in this study, we successfully prepared a new injectable conductive hydrogel using SAO and NH_2_-gelatine-PANI as raw materials and applied this hydrogel as the carrier of stem cells and DPL in the treatment of spinal cord injury.

First, we studied the physical properties of the hydrogels. It was found that the hydrogels could change from sol to gel in a short time through the vial tilt test, and the hydrogels could effectively adhere to the spinal cord tissue (Fig. 1). This property of the NGP hydrogels prevents the hydrogels from deviating due to the activity of the rat. It also ensures that no cavities are created after injection of the hydrogels, which facilitates rapid nerve growth. The conductivity measurement results show that the conductivity of the NGP hydrogels is 2.28 S/m (Fig. 2). Previous studies have shown that electroactive materials are beneficial to the proliferation and differentiation of nerve cells [34, 35]. Furthermore, conductive hydrogels can simulate the physiological environment of electroactive tissues and regulate cell adhesion and growth. The NGP hydrogels prepared by us showed very suitable electrical conductivity (1–10 S/m), which was recognized in previous studies, making them a suitable candidate material for spinal cord nerve repair [36]. In terms of biocompatibility, we found that NGP hydrogels showed no obvious internal toxicity or pathological changes after implantation, and hydrogels can be absorbed by the body in a short time. Some studies have found that polyaniline has certain cytotoxicity and can cause mild inflammatory reactions [37]. This is mainly due to the residues of the polyaniline precursor aniline. In addition, the molecular weight of polyaniline also affects its biocompatibility, and studies have found that the higher the molecular weight of polyaniline, the lower the toxicity [38]. Furthermore, the poor solubility and non-degradability of polyaniline have been the disadvantages that hinder its wide application. In this study, the biotoxicity of polyaniline was reduced by grafting polyaniline onto the main chain of amino gelatin. Many studies have shown that combining polyaniline with other natural polymers can effectively reduce the toxicity of polyaniline [26]. In addition, the biodegradability of conductive polyaniline was improved by grafting polyaniline on gelatin surface. As gelatin gradually degrades, polyaniline gradually breaks down into small molecules and is eliminated through the circulatory system. The HE staining results showed that there was only a slight inflammatory reaction and inflammatory cell aggregation in subcutaneous implantation sites, indicating that NGP hydrogels have good biocompatibility and can be safely applied in the body. By studying the physical and chemical properties of NGP hydrogels, we found that NGP hydrogels have good biocompatibility, tissue adhesion, self-repair and appropriate electrical conductivity, which preliminarily verified the feasibility of NGP hydrogels as carriers of NSCs and DPL.

A major objective of this study is to achieve long-term local release and persistence of therapeutic drugs at the site of spinal cord injury using NGP hydrogels. We observed a sustained DPL release profile from the NGP hydrogels for up to 8 days, and the release pattern had no initial burst effect. As expected, the NGP hydrogels with a denser network structure sustained the release of the incorporated DPL, and these results indicated that the NGP hydrogel matrix has the ability to sustain the release of therapeutics for a long period of time at body temperature. We evaluated the effects of NGP hydrogels and DPL on cell proliferation and differentiation using NSCs in vitro, which laid a foundation for further in vivo experiments using hydrogel loaded with DPL and NSCs. First, we tested the cytotoxicity of NGP hydrogels using live and dead cell staining experiments (Fig. 5). The results showed that a large number of NSCs cultured on NGP hydrogels survived, with a small number of dead cells, and live cells adhered well to the NGP hydrogels. This shows that our NGP hydrogels have low cytotoxicity and good cytocompatibility. Our next objective was to explore the proliferation and differentiation of NSCs when NGP hydrogels and DPL were applied individually and in combination, and the results of the CCK-8 experiment showed that NGP hydrogels promoted the proliferation of NSCs to a certain extent, while DPL did not. Furthermore, quantitative analysis of the length of nerve axons showed that the NGP hydrogels themselves promoted the growth and extension of newborn nerve axons, which were not possessed by DPL. Studies have shown that hyaluronic acid is important for maintaining and producing functional synapses and neurons [39, 40]. The main component of the hyaluronic acid receptor on the cell surface is CD44, which can mediate cell matrix adhesion, cell migration and cell signalling [41, 42]. Some experiments have also found that electroactive polymers (such as polyaniline, polypyrrole, polythiophene, etc.) can support the activity, proliferation and differentiation of NSCs cultured on them [43–45]. These reasons may be the reason why NGP hydrogels can promote NSC proliferation. Cell immunofluorescence staining and RT‒PCR experiments showed that NGP hydrogels could promote the differentiation of NSCs into new neurons and oligodendrocytes, but the promotion effect was significantly weaker than that of the drug DPL (Figs. 6 and 7). Meanwhile, NGP hydrogels and drug DPL can inhibit the differentiation of NSCs into astrocytes, and this effect is most obvious when the two are combined. DPL is a selective acetylcholinesterase inhibitor; however, its related pharmacological mechanism is not limited to this [46]. By inhibiting signal transducer and activator of transcription 3 (STAT3) and activating SMAD1/5/9 and their downstream target genes, DPL can increase the differentiation of oligodendrocytes, resulting in reduced differentiation of NSCs into astrocytes [47]. Several studies have also demonstrated that DPL can stimulate oligodendrocyte differentiation and myelin-related gene expression through activation of nicotinic acetylcholine receptors [48]. Therefore, the above cell experiment results show that the NGP hydrogel is an ideal platform suitable for the growth of NSCs, while the inclusion of DPL to direct NSC differentiation can further improve its therapeutic effect.

The reconstruction of neural circuits and the recovery of motor function after spinal cord injury are difficult problems. Biomaterials can reattach to the damaged site but cannot replace cells lost during SCI. Stem cell transplantation can replenish cells, but it cannot imitate the complex structure of the spinal cord, and the transplanted cells cannot survive in the harsh postinjury microenvironment. Therefore, combining biomaterials with stem cells has been identified as an emerging and intriguing strategy for promoting SCI repair. Furthermore, considering that the differentiation of stem cells such as NSCs into benign cells such as new neurons and oligodendrocytes has certain limitations, the use of biomaterials loaded with drugs as inducers of directed differentiation can further improve the therapeutic effect. Based on the above ideas, the ultimate purpose of this study is to use the combination of NGP hydrogels, NSCs, and DPL to treat spinal cord injury. After 8 weeks, the recovery of motor function was assessed by the BBB score (Fig. 8A). The BBB results showed that the NGP + NSC + DPL group achieved the highest score of 10 after 6 weeks, indicating that NGP + NSCs + DPL can significantly promote the recovery of motor function after spinal cord injury. Electromyography results suggested that the NGP + NSC + DPL group had the most similar amplitude to the normal rats, which is consistent with the BBB results (Fig. 8C). After spinal cord injury, cystic cavities of different sizes appeared in the injured area. These cavities have adverse effects on the regeneration of the spinal cord. Through HE staining, we found that the NGP + NSC + DPL group could significantly reduce the cavity area of the injured area, indicating that more cells were generated in the injured area (Fig. 9B and D). Myelin protein is the main component of axons and myelin sheaths, and LFB is a specific stain for myelin protein. The amount of myelin regeneration was greater in the NGP + NSC + DPL group than in any of the other groups (Fig. 9C and E). These results suggest that NGP + NSC + DPL treatment can significantly promote myelination in the damaged area.

Subsequently, Tuj-1, NF200 and C56 were used to stain and label newborn neurons, axons and glial scars, respectively (Fig. 10). The number of neurons and axon area in the two groups of transplanted NSCs were significantly higher than those in the control group and the NGP group, and the NGP + DPL + NSCs group was significantly higher than that in the NGP + NSCs group. These results indicate that NSCs transplanted by NGP hydrogel can successfully differentiate into neurons and generate axons for normal physiological activities, and this effect can be further expanded by using DPL as an inducer of directed differentiation. Hyperplastic astrocytes form a glial scar border surrounding cystic cavities, which hinders growth and the connection of axons between injured tissue and healthy tissue [49]. In this study, severe scar hyperplasia was found in the control group, while the CS56-positive area in the NGP + DPL + NSC group was significantly smaller than that in the other three groups, which indicated that the combination of NGP hydrogel, NSCs, and DPL could minimize the differentiation of NSCs into astrocytes, thus inhibiting the formation of glial scars and contributing to nerve regeneration. According to the above results, the combination of NGP + DPL + NSCs has a very obvious effect on the recovery of motor function and nerve conduction function. At the same time, this treatment strategy significantly increased the myelin sheath area and the number of new neurons and axons and minimized the formation of cystic cavities and glial scars. Therefore, the therapeutic strategy of NGP + DPL + NSCs for spinal cord injury is feasible and effective.

## Conclusions

In this study, we prepared an injectable conductive hydrogel NGP using SAO and NH_2_-Gel-PANI as raw materials and used it for loading NSCs and DPL. NGP hydrogels showed good electrical activity and biocompatibility and promoted NSC proliferation in vitro. NGP hydrogels have a good capacity for sustained release of drugs such as DPL, which is conducive to the differentiation of NSCs and the formation of new tissues. Furthermore, both cell proliferation and cell differentiation tests showed that the prepared hydrogels had little cytotoxic effect on NSCs, and the hydrogels loaded with DPL could further enhance the activities of cell proliferation and differentiation. When NGP hydrogel loaded with NSCs and DPL was implanted into a complete SCI rat model, we observed that the transplanted NSCs showed significant neuronal differentiation and integration in the injured area and reduced the formation of glial scars around the injured area. Furthermore, SCI rats in the NGP + DPL + NSC group showed the best improvement in motor function. In conclusion, the combination of NGP hydrogels, NSCs, and DPL provides a promising treatment option for spinal cord injury.

## Supplementary Information

**Additional file 1.**

## Data Availability

All data generated or analysed during this study are included in this published article [and its supplementary information files].

## Abbreviations

SCI: Spinal cord injury
NGP: Injectable electroactive hydrogel
SAO: Sodium hyaluronate oxide
NSCs: Neural stem cells
DPL: Donepezil
PANI: Polyaniline
PPy: Polypyrrole
PTH: Polythiophene
SEM: Scanning electronmicroscope
FTIR: Fourier transform infrared spectrometer
