# Supplementary material for: An injectable, self-healing, electroconductive hydrogel loaded with neural stem cells and donepezil for enhancing local therapy effect of spinal cord injury
Source: J Biol Eng. 2023 Jul 24;17:48. doi: 10.1186/s13036-023-00368-2 (PMC10367392; doi:10.1186/s13036-023-00368-2)

**Support information for**

**An injectable, self-healing, electroconductive hydrogel loaded with neural stem cells and donepezil for enhancing local therapy effect of spinal cord injury**

Tiemei Liu^1^, Hongru Li^2^, Xiaoqian Cui^3^, Qiang Zhang^2^, Zhiping Qi^2*^, Xiaoyu Yang^1,2*^

^1^ Department of Blood Transfusion, China-Japan Union Hospital of Jilin University, Changchun 130033, China

^2^ Department of Orthopaedic Surgery, The Second Hospital of Jilin University, Changchun 130041, China

^3^ Department of Emergency and Critical Care, The Second Hospital of Jilin University, Changchun 130041, PR China

*** Corresponding author:**

Xiaoyu Yang, Email: yangxiaoy@jlu.edu.cn

Zhiping Qi, E-mail: [qizp@jlu.edu.cn](mailto:qizp@jlu.edu.cn)

**Other author：**

Tiemei Liu, Email: [ltm@jlu.edu.cn](mailto:ltm@mails.jlu.edu.cn)

Hongru Li, Email: lhr1223@139.com

Xiaoqian Cui, Email: 2723497226@qq.com

Qiang Zhang, Email: azhangqiang_1988@126.com

**Supplementary Figures**

**Fig. S1** **(A) Ultraviolet absorption spectra of DPL at different concentrations. (B) DPL standard curve.**


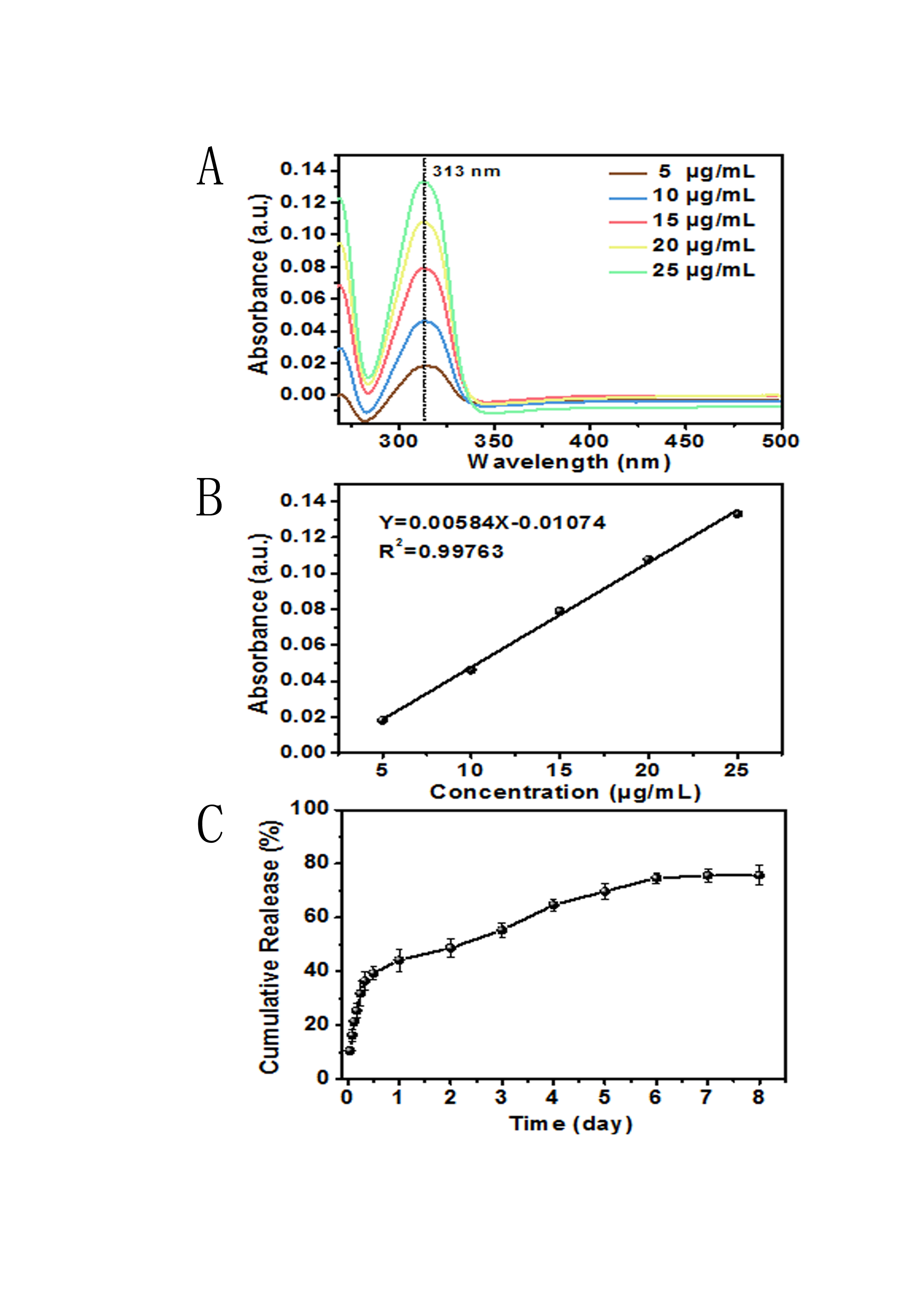

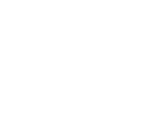

Supplement: Supplementary file 1 — Additional file 1. [file 13036_2023_368_MOESM1_ESM.doc]
